# Hyperbaric oxygen treatment reveals spatiotemporal OXPHOS plasticity in the porcine heart

**DOI:** 10.1093/pnasnexus/pgae210

**Published:** 2024-05-30

**Authors:** Juliana Heidler, Alfredo Cabrera-Orefice, Ilka Wittig, Estelle Heyne, Jan-Niklas Tomczak, Bjoern Petersen, Dirk Henze, Jaakko L O Pohjoismäki, Marten Szibor

**Affiliations:** Functional Proteomics, Institute of Cardiovascular Physiology, Faculty of Medicine, Goethe University Frankfurt, 60590 Frankfurt am Main, Germany; Experimental Vascular Surgery, University Clinic of Vascular Surgery, Innsbruck Medical University, 6020 Innsbruck, Austria; Functional Proteomics, Institute of Cardiovascular Physiology, Faculty of Medicine, Goethe University Frankfurt, 60590 Frankfurt am Main, Germany; Functional Proteomics, Institute of Cardiovascular Physiology, Faculty of Medicine, Goethe University Frankfurt, 60590 Frankfurt am Main, Germany; Department of Cardiothoracic Surgery, Center for Sepsis Control and Care (CSCC), Jena University Hospital, Friedrich Schiller University of Jena, 07747 Jena, Germany; Functional Proteomics, Institute of Cardiovascular Physiology, Faculty of Medicine, Goethe University Frankfurt, 60590 Frankfurt am Main, Germany; Institute of Farm Animal Genetics, Friedrich-Loeffler-Institute (FLI), 31535 Mariensee, Germany; Praxis für Anästhesiologie, Dr. Henze & Partner GbR, 06116 Halle (Saale), Germany; Department of Environmental and Biological Sciences, University of Eastern Finland, 80101 Joensuu, Finland; Department of Cardiothoracic Surgery, Center for Sepsis Control and Care (CSCC), Jena University Hospital, Friedrich Schiller University of Jena, 07747 Jena, Germany; Faculty of Medicine and Health Technology, Tampere University, 33014 Tampere, Finland

**Keywords:** pig, heart, mitochondria, complexome, hyperbaric oxygen

## Abstract

Cardiomyocytes meet their high ATP demand almost exclusively by oxidative phosphorylation (OXPHOS). Adequate oxygen supply is an essential prerequisite to keep OXPHOS operational. At least two spatially distinct mitochondrial subpopulations facilitate OXPHOS in cardiomyocytes, i.e. subsarcolemmal (SSM) and interfibrillar mitochondria (IFM). Their intracellular localization below the sarcolemma or buried deep between the sarcomeres suggests different oxygen availability. Here, we studied SSM and IFM isolated from piglet hearts and found significantly lower activities of electron transport chain enzymes and F_1_F_O_-ATP synthase in IFM, indicative for compromised energy metabolism. To test the contribution of oxygen availability to this outcome, we ventilated piglets under hyperbaric hyperoxic (HBO) conditions for 240 min. HBO treatment raised OXPHOS enzyme activities in IFM to the level of SSM. Complexome profiling analysis revealed that a high proportion of the F_1_F_O_-ATP synthase in the IFM was in a disassembled state prior to the HBO treatment. Upon increased oxygen availability, the enzyme was found to be largely assembled, which may account for the observed increase in OXPHOS complex activities. Although HBO also induced transcription of genes involved in mitochondrial biogenesis, a full proteome analysis revealed only minimal alterations, meaning that HBO-mediated tissue remodeling is an unlikely cause for the observed differences in OXPHOS. We conclude that a previously unrecognized oxygen-regulated mechanism endows cardiac OXPHOS with spatiotemporal plasticity that may underlie the enormous metabolic and contractile adaptability of the heart.

**Significance Statement**

The heart relies on oxidative phosphorylation (OXPHOS) for energy supply for organ homeostasis and contractility. However, the size of cardiomyocytes conceivably leads to intracellular oxygen gradients making subcellular mechanisms to regulate OXPHOS a vital necessity. In spatially distinct mitochondrial subpopulations from piglet hearts, we uncovered an oxygen-controlled mechanism that enables OXPHOS adaptation in a spatiotemporal manner. This mechanism may underlie the enormous metabolic and contractile adaptability of the heart and should thus be a subject of further basic and clinical research.

## Introduction

The adult human heart has an enormous energy demand. It turns over up to 6 kg ATP per day to maintain contraction, ion transport across membranes and organ homeostasis (1). Yet corresponding ATP stores are lacking, so that a continuous and on-demand ATP supply is indispensable to avoid organ dysfunction (2–4). In cardiomyocytes, ATP is almost exclusively provided by mitochondrial oxidative phosphorylation (OXPHOS), which consists of the respiratory chain (or electron transport chain [ETC]) and the F_1_F_O_-ATPase synthase. The ETC is composed of four high-molecular mass protein complexes (cI–cIV) and two electron carriers (coenzyme Q and cytochrome *c*) (5) facilitating a series of redox reactions. The electron transfer through the ETC is coupled to proton translocation across the inner mitochondrial membrane thus producing an electrochemical gradient (or proton motive force [pmf]), which is itself the main driving force for ATP generation by the F_1_F_O_-ATP synthase (or cV). Oxygen is the final electron acceptor in this series of redox reactions, which explains why oxidative metabolism and ATP production are intrinsically coupled (6) and why functioning of cardiomyocytes is critically dependent on adequate tissue oxygenation.

Oxygen availability, however, can be very heterogeneous particularly in large and highly structured cells such as cardiomyocytes. Ironically, OXPHOS machineries located within layers of mitochondria consumes oxygen while it diffuses through the cell, thereby conceivably creating a gradient that may limit the availability of oxygen in the contractile center of cardiomyocytes. Indeed, previous work in uncoupled and paced rat cardiomyocytes visualized radial oxygen gradients that rendered the cellular center hypoxic (7–10). This suggests that ATP production may become limited at times and at the anatomical site of greatest demand, which may eventually worsen under conditions of increased workload or cardiac hypertrophy. Tightly controlled mechanisms must exist to adjust OXPHOS in a spatiotemporal manner. Recently, the ETC was identified as an oxygen sensor (11–13) leading us to hypothesize that the ETC might sense oxygen levels on the subcellular level within cardiomyocytes. To test this assumption, we took advantage of methods to isolate different mitochondrial subpopulations, i.e. subsarcolemmal (SSM) and interfibrillar mitochondria (IFM) (14). Importantly, their subcellular localization within cardiomyocytes underneath the sarcolemma or buried deep between the sarcomeres conceivably predicts different oxygen availability.

Here, we found that activities and complex composition of ETC enzymes and the F_1_F_O_-ATP synthase differ in a subpopulation-specific manner. To test for the role of oxygen, we applied hyperbaric hyperoxic (HBO) conditions, which adjusted all activities in IFM to the level of SSM. Particularly puzzling was an observed oxygen-dependent plasticity of F_1_F_O_-ATP synthase in composition and activity specifically in IFM indicative for the existence of mechanisms that enable a dynamic switch between states of energy saving and energy production. Essentially, this mechanism may allow a spatiotemporal adaptation of cardiac OXPHOS and contribute to the enormous metabolic and contractile flexibility of the heart.

## Results

### Mitochondrial enzyme activities exhibit spatiotemporal differences

**Fig. 1.**
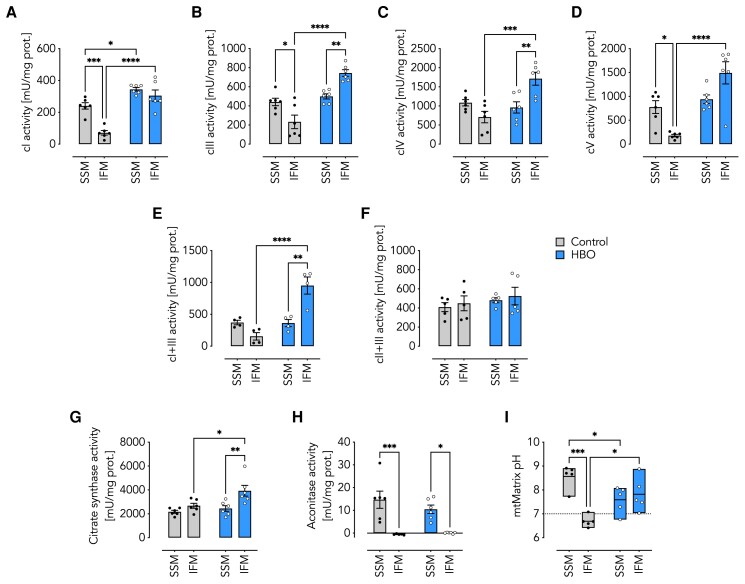
Mitochondrial enzyme activities and matrix pH. A–H) Specific activities (milliunits per mg protein) of OXPHOS enzymes in SSM and IFM from control and HBO-treated piglets as indicated. A) Complex I (cI) activity (*n* = 6). B) Complex III (cIII) activity (*n* = 6). C) Complex IV (cIV) activity (*n* = 6). D) Complex V (cV, ATPase) activity (*n* = 6). E) Complexes I + III (cI + III) activity (*n* = 4). F) Complexes II + III (cII + III) activity (*n* = 5). G) Citrate synthase activity (*n* = 6). H) Aconitase activity (*n* ≥ 5). I) Matrix pH estimated using the pH-sensitive fluorescent dye BCECF (*n* = 5). A–H) Data are shown as the mean ± SEM (error bars) from the number of independent experiments as indicated. I) Data are shown as floating bars (min to max) with line at mean. *P < 0.05, **P < 0.01, ***P < 0.001, ****P < 0.0001 for comparisons as indicated by two-way ANOVA with Tukey's multiple comparisons post hoc test. SSM, subsarcolemmal mitochondria; IFM, interfibrillar mitochondria; control, piglets ventilated at ambient conditions; HBO, piglets ventilated at hyperbaric hyperoxic conditions.

To elucidate a potential role of subcellular oxygen availability in local OXPHOS adaptation, we studied two distinct mitochondrial subpopulations in the left cardiac ventricle, i.e. SSM and IFM (14), and chose the rather elaborate piglet model because its cardiovascular system is, in terms of physiology and anatomy, similar to that of humans (15). In a standardized procedure, anesthetized piglets were ventilated under normobaric conditions with a FiO_2_ (fraction of inspired oxygen concentration) of 0.3 (hereafter referred to as control, a detailed description of all interventions is given in “Materials and Methods” section and Fig. S1). After termination of experiments, hearts were excised and SSM and IFM were quickly isolated. In freshly isolated mitochondria, we observed significantly lower activities of different OXPHOS enzymes in IFM compared with SSM namely respiratory complex I (cI, Fig. 1A), complex III (cIII, Fig. 1B), and complex V (cV or F_1_F_O_-ATP synthase, Fig. 1D). Other mitochondrial enzymes appeared unaffected, or the observed changes did not reach statistical significance such as complex IV (cIV, Fig. 1C) also measured in freshly isolated samples, or the combined activity of complex I + III (cI + III, Fig. 1E) and complex II + III (cII + III, Fig. 1F) measured in frozen-thawed samples. Since, the activity of OXPHOS enzymes inevitably affects upstream metabolic circuits such as the tricarboxylic acid (TCA or Krebs) cycle, we set out to test the activity of citrate synthase, the enzyme that catalyzes the first step of TCA by forming a carbon–carbon bond between oxaloacetate and acetyl-CoA yielding citrate, as well as aconitase (ACO), an enzyme that catalyzes the isomerization of citrate to isocitrate, in freshly isolated and frozen-thawed samples, respectively. We found distinct activity patterns for some of the Krebs cycle enzymes. While citrate synthase activity was similar in SSM and IFM (Fig. 1G), ACO was reliably measurable in SSM but only at the detection threshold in IFM (Fig. 1H).

### HBO ventilation activates IFM

The significant decrease in key OXPHOS activities and the ambiguous results regarding Krebs cycle enzymes came as a surprise and appeared somewhat counterintuitive since OXPHOS activities were previously reported to be higher in IFM than in SSM (16–20). This raised concerns as to whether using the piglet model was indeed an appropriate approach for studying OXPHOS adaptations as seen in the human heart. It also appeared dubious how ATP levels required for contractility could possibly be maintained in the compartments near to IFM. We reasoned that the observed differences in enzyme activities may in fact be due to their distinct cellular localization and reflect different oxygen availabilities. If this hypothesis was correct, then the enzyme activities in IFM would adjust to SSM levels upon improved tissue oxygenation. Therefore, we decided to ventilate piglets under hyperbaric hyperoxic conditions (hereafter referred to as HBO), a treatment previously shown to improve cardiac tissue oxygenation and conferring cardioprotective effects under conditions such as carbon monoxide poisoning and ischemia (21–25). Briefly, anesthetized piglets of the HBO group were ventilated in a pressure chamber at 2.4 bar with a FiO_2_ of 1.0 for 240 min under otherwise identical conditions as control piglets (Fig. S2). HBO ventilation markedly increased most OXPHOS enzyme activities in IFM (Fig. 1A–E and G) with two exceptions, i.e. cII + III (Fig. 1F), which showed no difference, and ACO (Fig. 1H), which remained near the detection threshold.

Since electron flux through the ETC is a prerequisite to build-up a proton gradient for ATP generation, we next set out to assess the mitochondrial matrix pH as an indirect measurement of the *pmf* value. Using the pH-sensitive fluorescent dye BCECF (acetoxymethyl ester of 2 h,7h-bis-(2-carboxyethyl)-5-carboxyfluorescein) (26) on freeze-thawed SSM and IFM, we found that the matrix pH of control IFM decreased to ∼7, indicating a low *pmf* value. This result was consistent with decreased activities of both ETC complexes and F_1_F_O_-ATP synthase. HBO ventilation leveled the matrix pH of IFM to the more alkaline value of SSM (Fig. 1I).

### F_1_F_O_-ATP synthase holoenzyme dynamically assembles following tissue oxygenation

**Fig. 2.**
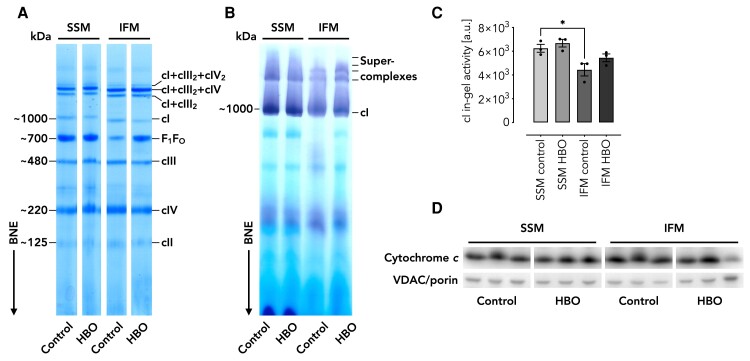
Migration patterns of OXPHOS complexes of digitonin-solubilized mitochondria and cI in-gel activity stains. A) Gel showing Coomassie stain of digitonin-solubilized SSM and IFM from control and HBO-treated piglets as indicated separated by blue-native electrophoresis (BNE). B) Gel showing catalytic in-gel staining of complex I (cI) from digitonin-solubilized and BNE-separated mitochondria as shown in A). C) Quantification of cI in-gel activities (*n* = 3) as shown in B). For the gel showing all replicates used for quantification please refer to Fig. S3A. Data are shown as the mean ± SEM (error bars). *P < 0.05 for comparison as indicated by one-way ANOVA with Tukey's multiple comparisons post hoc test. D) Western blots indicating cytochrome *c* and outer membrane protein VDAC/porin loading. Note: The gels in A) and D) have been cropped and arranged according to the loading in B) for easier comprehension. SSM, subsarcolemmal mitochondria; IFM, interfibrillar mitochondria; control, piglets ventilated at ambient conditions; HBO, piglets ventilated at hyperbaric hyperoxic conditions; cI–cIV, respiratory complexes I–IV.

We next sought to test if the decreased values for OXPHOS enzyme activities in IFM controls, particularly the F_1_F_O_-ATP synthase, were due to a decrease in holoenzyme quantities or if enzyme activities were impaired. To separate OXPHOS complexes of SSM and IFM, we performed blue-native electrophoresis (BNE) (27, 28) and used digitonin to solubilize mitochondria as this mild detergent preserves respiratory supercomplexes (SCs). Following separation by BNE, we stained the gels with Coomassie blue dye to visualize individual complexes and complex assemblies and found that all OXPHOS enzymes were present in control IFM with the F_1_F_O_-ATP synthase being markedly decreased (Fig. 2A). Next, we sought to assess whether the enzyme assemblies were catalytically active and applied in-gel assays (29) visualizing catalytic activities for NADH oxidation (reflecting cI activity) (Figs. 2B and C, S3A). The catalytic in-gel staining corroborated our spectrophotometric measurements qualitatively. We also tested whether total mitochondrial loading confounded the result and tested this by western blotting against the mitochondrial marker proteins cytochrome *c* and VDAC/porin (Fig. 2D), which revealed no obvious difference. We concluded that tissue oxygenation must be sufficient to specifically adjust the composition and activity of OXPHOS complexes spatially and temporally in IFM.

We then wanted to investigate whether the F_1_F_O_-ATP synthase is similarly regulated in an oxygen-dependent manner and used n-dodecyl-β-D-maltoside (DDM)-solubilized mitochondria, a treatment that resolves individual complexes and does not provide information about their supramolecular organization. This is a suitable approach since the F_1_F_O_-ATP synthase does not integrate into respiratory chain SCs. As outlined for digitonin-solubilized mitochondria, BNE was applied, and complexes were visualized with Coomassie staining (Fig. S3B). The outcome was similar to digitonin solubilization of mitochondria although the F_1_F_O_-ATP synthase content appeared to be even lower. Next, we tested by in-gel staining for ATPase activity (reflecting F_1_F_O_-ATP synthase quantity) and found that the activity was below the detection threshold in control IFM, but it increased markedly upon HBO ventilation (Fig. S3C).

To confirm that the bands emerging in IFM upon HBO ventilation in both the Coomassie and activity staining indeed represents the F_1_F_O_-ATP synthase, we resolved the native gel slices in a second dimension by using tricine-SDS polyacrylamide gel electrophoresis (30) and visualized individual subunits thereafter by silver stain (Fig. S3D). This approach revealed the typical separation pattern for the F_1_F_O_-ATP synthase subunits, thus confirming its original assignment. Unexpectedly, we observed additional bands, which were identified by mass spectrometry (MS) as part of the beta subunit. Since cleavage products of F_1_F_O_-ATP synthase subunits were previously described to appear upon stress induced by reactive oxygen species (ROS) (31), we further validated the existence of fragments by western blot (Fig. S3E and F). Caution is warranted, however, as based on the data presented, we cannot conclusively distinguish between ROS-mediated cleavage products as a result of hypoxia or HBO-induced ROS stress, the presence of disproportionate OXPHOS complex assemblies as a result of HBO-mediated reconstitution and/or degradation of exposed subunits due to the isolation procedure in which the protease Nagarse is used to extract IFM.

### Upregulation of OXPHOS by HBO is partially achieved at the transcription level

**Fig. 3.**
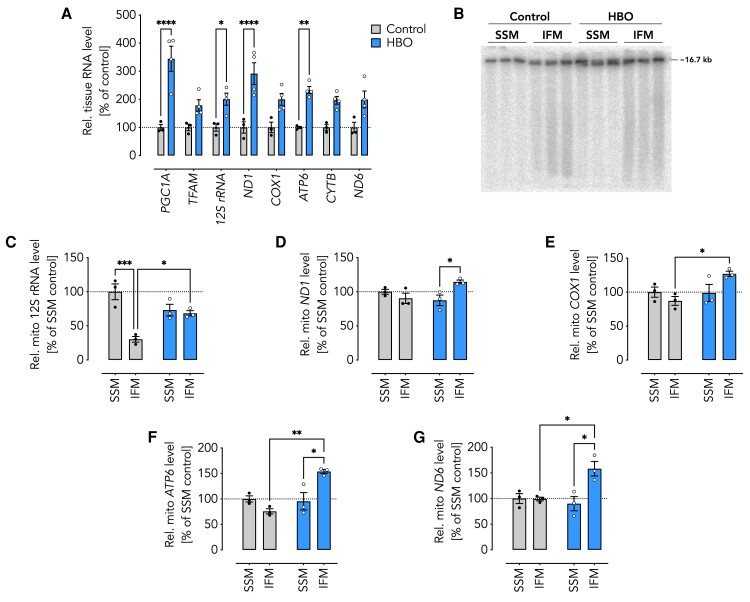
Level of selected transcripts and mtDNA integrity in porcine heart tissue, SSM and IFM. A) Relative transcript level (% of control) in heart tissue from control (*n* = 3) and HBO-treated (*n* = 4) piglets as indicated. B) Southern blot probed against ND6. C–G) Relative level (% of SSM control) of selected transcripts in SSM and IFM (*n* = 3) from control and HBO-treated piglets as indicated. C) *12S rRNA* (primary transcript 1); D) *ND1*, E) *COX1*, F) *ATP6* (primary transcript 2), and G) *ND6* (primary transcript 3). Data are shown as the mean ± SEM (error bars) from the number of independent experiments as indicated. *P < 0.05, **P < 0.01, ***P < 0.001, ****P < 0.0001 for comparisons as indicated by two-way ANOVA with Šídák's multiple comparisons post hoc test A) and Tukey's multiple comparisons post hoc test C–G). SSM, subsarcolemmal mitochondria; IFM, interfibrillar mitochondria; control, piglets ventilated at ambient conditions; HBO, piglets ventilated at hyperbaric hyperoxic conditions.

To determine how HBO may induce OXPHOS enzyme activities, we assessed transcript levels by reverse transcription (RT) quantitative polymerase chain reaction (qPCR). RT-qPCR analysis of RNA isolated from left ventricular tissue samples revealed significantly increased levels of two key regulators of mitochondrial biogenesis in the HBO group compared to control, i.e. *PPARGC1A* (*PGC1A*) and *TFAM*, as well as several transcripts encoded on the mtDNA (Fig. 3A). We next sought to identify the source of the increased mitochondrial transcripts. Since mtDNA is particularly susceptible to ROS damage, which affects mitochondrial transcription and gene expression, we checked mtDNA integrity. Southern blot analysis, however, revealed no evidence of gross degradation or the appearance of cleavage products (Fig. 3B). RT-qPCR analysis of RNA isolated from SSM and IFM revealed that the HBO-associated increase in mitochondrial transcripts was largely confined to the IFM subpopulation (Fig. 3C–G). We conclude that the induction of mitochondrial biogenesis by a concerted increase in nuclear and mitochondrial gene expression may be in part responsible for the observed restoration of enzyme activities.

### Holoenzyme assembly utilizes preserved protein subunits and partially assembled subcomplexes

**Fig. 4.**
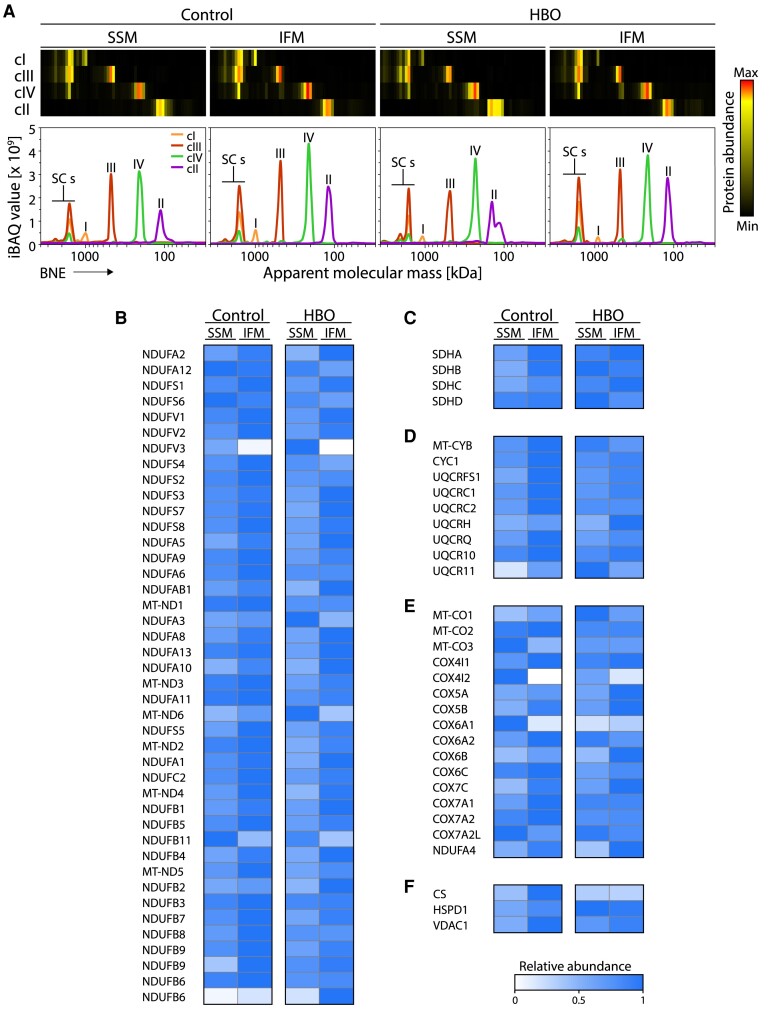
Complexome profiling of ETC complexes cI–IV. A) SSM and IFM were solubilized using digitonin and separated by blue-native electrophoresis (BNE) followed by quantitative mass spectrometry (MS) analysis. Protein abundance profiles of each respiratory complex were generated by averaging the intensity-based absolute quantification (iBAQ) values of all their individual subunits identified by MS. Resultant profiles are illustrated as heatmaps and 2D profile plots against the apparent molecular mass. B–F) MS quantification of individual subunits of the ETC complexes from control and HBO-treated piglets. Abundance was calculated from complexome profiles as the area under the curve (AUC) in the range where mature complex I (cI) B), complex II (cII) C), complex III (cIII) D), and complex IV (cIV) E) were identified. Data were normalized to the maximal value found across the samples. F) Relative abundances of three representative household mitochondrial proteins for reference. SSM, subsarcolemmal mitochondria; IFM, interfibrillar mitochondria; control, piglets ventilated at ambient conditions; HBO, piglets ventilated at hyperbaric hyperoxic conditions; cI–cIV, OXPHOS complexes I–IV.

**Fig. 5.**
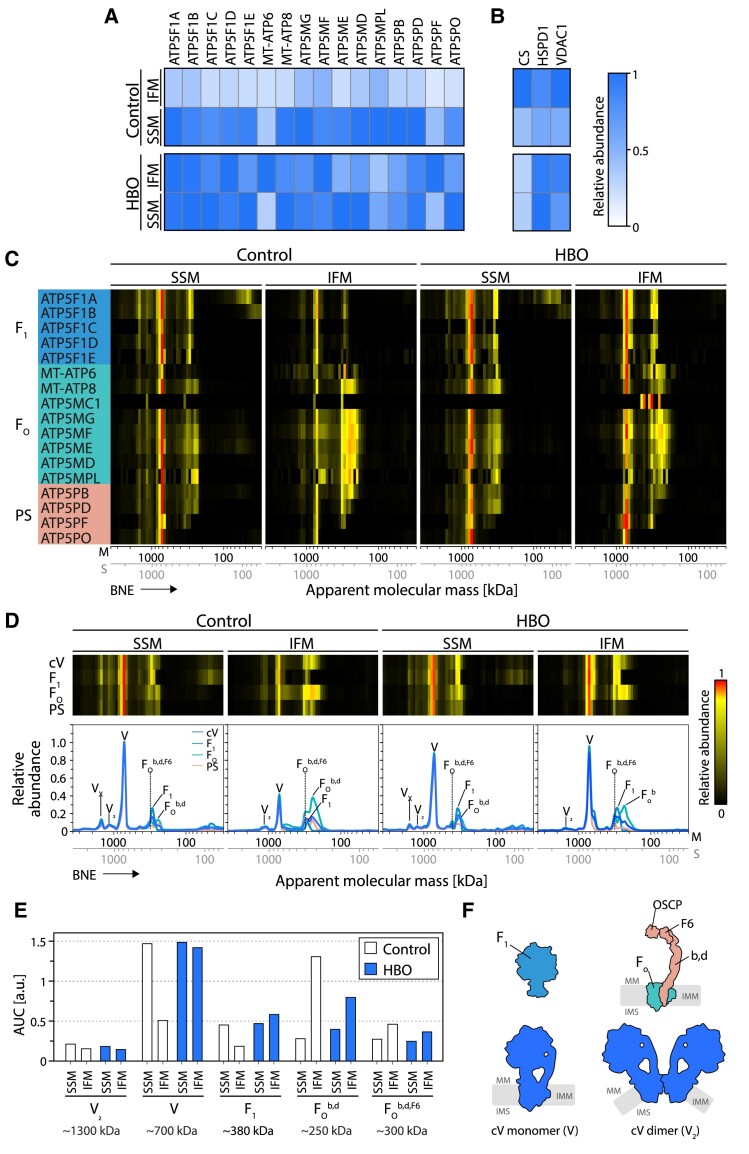
Complexome profiling analysis corroborates oxygen-dependent disassembly and reassembly of F_1_F_O_-ATP synthase in IFM. F_1_F_O_-ATP synthase abundance is largely decreased in control IFM, which returns to SSM levels upon HBO treatment. In control IFM, accumulation of membrane sub-assemblies as well as loss of soluble F_1_ subunit were observed. A) Mass spectrometry (MS) quantification of all ATP synthase (cV) subunits that were identified by complexome profiling. The iBAQ values of each subunit were normalized using their maximal values across profiles. Resultant relative abundance profiles are shown as heatmaps. B) Relative abundances of three representative household mitochondrial proteins for reference. C) Complexome profiles from the ATP synthase holoenzyme and the subunits that constitute each module are shown as heatmaps. The subunits are organized according to their structure location in the F_1_ subunit, F_O_ subunit and peripheral stalk (PS). D) Averaged complexome profiles of the individual ATP synthase modules. The observed fractions of cV oligomers, holoenzyme, and sub-assemblies are indicated next to their respective peaks. E) Abundance of each of the latter fractions was estimated by calculating the area under the curve (AUC). The apparent molecular masses where the peaks where observed are shown under the headers. F) Cartoon representations of the different modules and structure arrangements of ATP synthase (also see Fig. 7). The models were generated using the structure of porcine ATP synthase; PDB: 6J5I. The scales for apparent molecular mass are displayed for both membrane (M, upper scale) and soluble (S, lower scale) proteins. MM, mitochondrial matrix; IMM, inner mitochondrial membrane; IMS, intermembrane space; SSM, subsarcolemmal mitochondria; IFM, interfibrillar mitochondria; control, piglets ventilated at ambient conditions; HBO, piglets ventilated at hyperbaric hyperoxic conditions; cV, F_1_F_O_-ATP synthase.

Because HBO ventilation lasted 240 min only, we regarded it as rather unlikely that the observed recovery of OXPHOS activities was entirely dependent on de novo generation of subunits and assembly of complexes from scratch. This is particularly true for the F_1_F_O_-ATP synthase. We thus sought to test whether the OXPHOS complexes were already present in an inactive or partially assembled form in the inner membrane of mitochondria prior to HBO treatment. Digitonin-solubilized SSM and IFM were separated by BNE, and gel slices were further analyzed by complexome profiling, a method that identifies and quantifies proteins by MS and provides information on the assembly status of protein complexes (32, 33). We identified all ETC complexes in their regular stoichiometry. Complex cI predominantly associated in respiratory SCs together with cIII and cIV, that were increased ∼1.5-fold upon HBO treatment (Figs. 4A–F and S4A and B). Specifically, cII, cIII, and cIV were present mostly as individual holoenzymes at similar levels. Conversely, F_1_F_O_-ATP synthase showed remarkable differences in quantity and distribution in different subpopulations (Fig. 5A–D).

In control IFM, the amount of the fully assembled F_1_F_O_-ATP synthase was only ∼30% of that in SSM. While the soluble F_1_ component in control IFM was correspondingly lower ∼60%. Accumulation of sub-assemblies of the F_O_ segment containing peripheral stalk (PS) subunits b (ATP5PB) and d (ATP5PD), with or without subunit F6 (ATP5PF), was observed (Fig. 5E and F for diagrammatic summaries). No clear co-migration of known assembly factors such as TMEM70 was observed in the sub-assemblies. Complexome profiling analysis of HBO IFM showed an increased level of the F_1_F_O_-ATP synthase holoenzyme, now matching that of SSM, along with an increased level of free F_1_, although dimers were less altered. Together this indicates that preformed and/or preserved protein subunits and subcomplexes await holoenzyme assembly upon demand, and this assembly appears to be regulated by intracellular oxygen availability.

### Full tissue proteome analysis provides no evidence of adverse remodeling induced by HBO

**Fig. 6.**
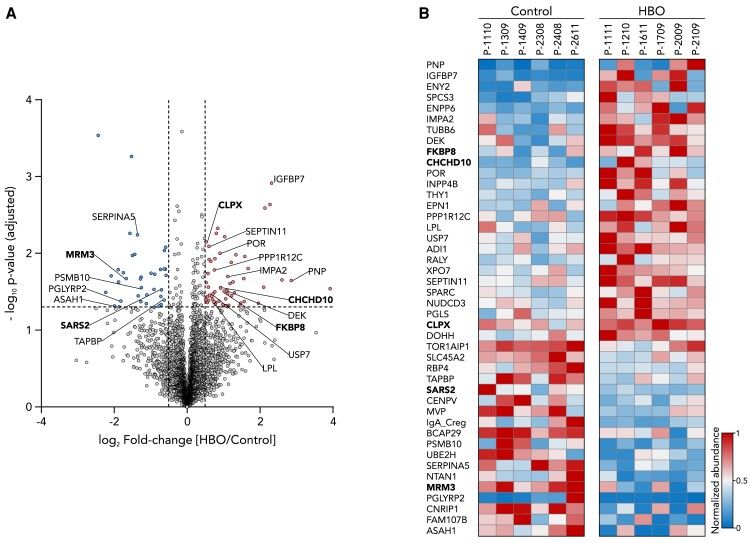
Proteome analysis of cardiac left ventricular tissue. A) Volcano plot visualization of full proteome analysis of control and HBO-treated cardiac tissue. Proteins that were significantly increased in HBO piglets are shown in red (right side), while proteins that were significantly diminished are shown in blue (left side). Mitochondrial protein hits are labeled in bold text. Significance thresholds: Fold change >1.5 and *P* < 0.05 (value adjusted) (*n* = 6 piglets per condition). B) Heatmap visualization of significant proteins identified. Data were normalized to the maximal value found across the samples. Mitochondrial protein hits are labeled in bold text (*n* = 6 piglets per condition).

To ensure that the observed differences are not primarily due to general remodeling, which may be a negative effect of, for instance, HBO-induced ROS damage, we performed a full tissue proteome analysis. Given the dramatic effects on transcription, protein expression and enzyme activities described for control IFM, our data show seemingly little change at the tissue level (Fig. 6A and B), which however supports the notion that protein subunits of disassembled OXPHOS complexes are preserved in mitochondrial membranes. Most notably, of the ∼4,500 proteins identified, only 95 were significantly altered. After exclusion of hits with less than two unique peptides and ≥10% of sequence coverage, only 44 proteins were differentially expressed, of which five were mitochondrial. There was no visible enrichment of proteins from specific molecular processes, yet there were few proteins involved in signaling, transport, and gene expression. The five mitochondrial protein hits are involved in membrane organization (CHCHD10), Ca^2+^-related pathways (FKBP8), proteostasis (CLPX), and RNA processing (SARS2, MRM3). However, none of these proteins were directly involved in OXPHOS biogenesis or mitochondrial energy metabolism. Altogether, our data suggest that the observed OXPHOS changes at the IFM are rather local thus ruling out adverse tissue remodeling as the main reason for the results.

## Discussion

**Fig. 7.**
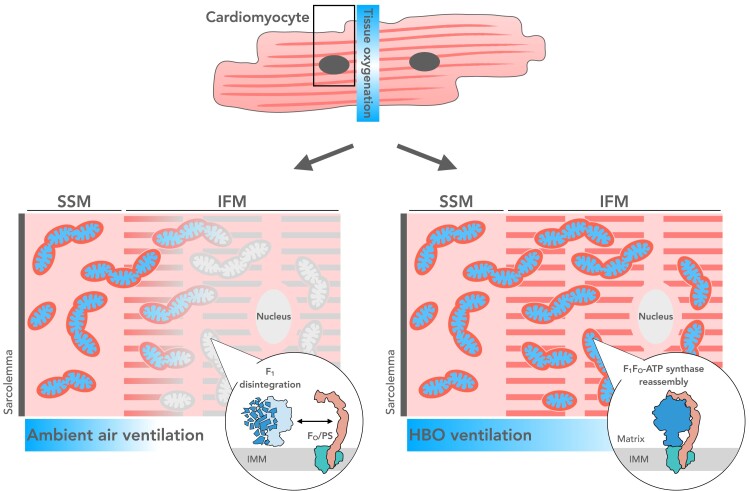
Graphical overview. Oxygen availability is a prerequisite for the oxidative phosphorylation (OXPHOS) system to be operational. Ventilation at ambient (control) or hyperbaric hyperoxic (HBO) conditions revealed in piglet heart subsarcolemmal (SSM) and intrafibrillar mitochondria (IFM) a dynamic OXPHOS plasticity regarding enzyme activities and composition. In control IFM, respiratory enzyme activities were lower compared with SSM, and F_1_F_O_-ATP synthase (or complex V [cV]) disassembled into individual protein subunits and subcomplexes. Notably, while the F_O_ subcomplex was largely intact, the catalytic F_1_ subcomplex disintegrated with its subunits still present in the inner mitochondrial membrane. The dynamic assembly and disassembly of OXPHOS complexes in an oxygen-controlled manner may be a mechanism underlying the enormous metabolic and contractile adaptability of the heart.

The heart covers its enormous energy demand almost exclusively by OXPHOS. Because cardiac ATP stores are essentially lacking, it turns over its entire cellular ATP pool in less than a minute (1). Thus, OXPHOS activity must adapt rapidly to demand to avoid organ failure (2–4). The dependence of OXPHOS on oxygen makes its abundance a vital necessity. Although we did not measure intracellular oxygen levels in vivo, our data suggest that the increased oxygen availability in the heart provided here experimentally by HBO ventilation leads to OXPHOS assembly and activation, with F_1_F_O_-ATP synthase and its catalytic subcomplex F_1_ being particularly affected (Fig. 7). Alterations in OXPHOS composition were measured at high resolution by complexome analysis (32, 33). To conclusively exclude uncharacterized off-target effects of HBO treatment and to avoid personal bias, we also performed quantitative tissue proteome analysis, which revealed that at tissue level only very few proteins were significantly altered with minimal fold changes. Moreover, the content of mitochondrial proteins, especially OXPHOS-related proteins, were barely changed, suggesting that enhanced tissue oxygenation, but not other unspecified phenomena, is most likely the trigger for the observed OXPHOS plasticity. Interestingly, this conclusion refines a prediction previously made (34). Here, we discuss our findings in the light of a clinically relevant question, namely how the heart remains metabolically stable, a prerequisite for continuous contractility, despite constantly changing workloads (35–37), and speculate how mammalian hearts may manage their impressive reserve capacity (38) without a need for anaerobic metabolism as seen in skeletal muscle (39).

Several modes of OXPHOS control are known (2, 3). ADP addition to properly oxygenated, coupled and energized (isolated) mitochondria, for instance, results in a burst of oxygen consumption and ATP production. This makes the P:O ratio (number of nanomoles of ATP produced per nanogram atom of oxygen consumed) a measure of OXPHOS coupling efficiency (40). Upon complete ADP conversion to ATP, mitochondrial respiration drops to a nonphosphorylating state even if oxygen and substrates are still present in abundance (41, 42). A feedback mechanism for ATP consumption solely relying on mitochondrial ADP sensing, however, seems incomplete, since in vivo studies in the canine heart revealed surprisingly constant levels of ATP, ADP, inorganic phosphate (P_i_) and creatine phosphate despite significant fluctuations in work intensity and oxygen consumption (35). This phenomenon was previously dubbed the “stability paradox” (36, 37). A central dilemma for intracellular oxygen distribution, however, is that cardiomyocytes are formidable entities with complex intracellular structures. In addition, oxygen needs to pass through several layers of respiring mitochondria to reach IFM. It is therefore not surprising that in the center of cardiomyocytes, hypoxia can be experimentally induced by uncoupling mitochondrial respiration (7–10). An ingenious idea of how contractile cells may circumvent dysfunction due to local lack of oxygen was previously brought forward, i.e. the formation of electrically competent mitochondrial networks (43, 44) or proton-conducting “cables” from well-oxygenated SSM to poorly oxygenated IFM. Such mechanism of proton conduction would in theory allow IFM to produce ATP even in the absence of oxygen, provided that SSM translocate enough protons. We cannot rule out the possibility that such a mechanism is essential for acute OXPHOS adaptations as demonstrated in cultured cardiomyocytes (10) or for anaerobic metabolism in skeletal muscle (39), but the fact that the ATP-generating capacity of the IFM was dramatically low under normoxic conditions suggests that proton conduction was not the underlying principle here. Conversely, our data support the notion that accurate oxygen-sensing mechanisms at the mitochondrial level must be in place such as previously demonstrated in cells of the pulmonary vasculature (11, 12) and carotid bodies (13).

Our data may also help explain another riddle debated for years, namely whether SSM and IFM are biochemically distinct and play distinct roles in health and disease (17, 18, 45–49) or are essentially the same (50, 51). Of note, the need for additional steps and protease digestion during isolation of IFM carries the risk of obscuring results and biasing its interpretation. In general, any type of mitochondrial isolation method results in a small percentage of organelles being damaged. Such damage is inevitably caused by mechanical interventions during homogenization. As a result, Nagarse, the protease used to isolate IFM, may have had access to varying amounts of broken mitochondrial membranes and exposed parts of some proteins in its native form. This may explain why such cleavage products migrated with the ATP synthase. Conversely, the additional bands of alpha and beta subunits of the F_1_F_O_-ATP synthase observed specifically in the IFM fraction may reflect local differences in oxygenation and oxidative stress (31) possibly due to reassembled but still disproportionate OXPHOS complexes or rapid oxidation of Krebs cycle intermediates that abnormally accumulate in the absence of oxygen. This assumption seems to be supported by the detection of an increased abundance of antioxidant proteins such as SOD2, especially in the IFM control (Fig. S4B). Underlying mechanisms such as the proteases involved, the signaling cascades activated and the consequences for enzyme activities, remained unexplored. Yet, given the reproducible effect of HBO on the organization and activity of OXPHOS complexes, we find it plausible that differences in local oxygen availability may account for the previously observed disparities between SSM and IFM in different disease models.

The observed decrease of IFM activities in the control group suggests that less ATP was produced by OXPHOS. Although not tested here, the core myofibrils may be more dependent on the ATP and creatine phosphate system fueled primarily by SSM. Alternatively, the changes observed in control IFM may be the consequence of substrate starvation. The latter has been already ruled out though by showing that cytoplasmic fatty acid-binding protein is abundantly present and therefore the supply of this main substrate may not be rate limiting (52). Notably, the healthy heart works best when there is an optimal ratio between fatty acids and glucose as substrates (53). In the hypertrophic heart, there is a shift toward increased glucose utilization at the expense of fatty acids, presumably because oxygen becomes scarce and fatty acid oxidation requires relatively more oxygen per molecule. It stands to reason that HBO allowed an increase in fatty acid oxidation relative to glucose, thereby restoring the healthy fatty acid to glucose ratio. An altered metabolism may also underlie the seemingly contradictory finding of the differential regulation of Krebs cycle enzymes. Our analysis of the complexome data unveiled that roughly half of the metabolic circuit is downregulated as ACO, isocitrate dehydrogenase, oxoglutarate dehydrogenase, and succinyl-CoA ligase had a lower abundance against the rest of the enzymes in control IFM. Upon HBO, the expression of these enzymes somewhat increased (Fig. S4B). The lower expression of ACO in control IFM was consistent with the very low activity values described above. The remaining difference between activity and protein content could be related to the fact that ACO is a redox sensor and very sensitive to ROS (54). In addition, the ACO reaction exhibits a long lag phase before it reaches a steady state. Although we ran the assays very carefully, we cannot completely rule out that the activity measured underestimates the in vivo value. We also compared the abundance of other major energy metabolism pathways in heart, namely beta-oxidation and ketone bodies oxidation (Fig. S4B). We found that the enzymes mediating such pathways were slightly more abundant in control IFM than in SSM. While HBO treatment did not have a major effect in SSM, most of the enzymes from the two pathways were visibly downregulated in IFM. Why exactly this happens in IFM is not straightforward to answer, but the expression pattern of proteins involved in the oxidation of ketone bodies/fatty acids, citrate transport, and alternative electron transfer pathways seems to indicate a “hypoxia-like” state of the control IFM. If this is true, the ETC flux could be considerably fed by electron carriers derived from metabolic pathways other than glucose and fatty acids oxidation. In this scenario, the export of citrate and fatty acids could serve to prevent excessive reduction of the NAD pool and formation of ROS. The disassembly of ATP synthase in IFM could thus be a mechanism to avoid local ATP depletion while allowing ATP produced by SSM to diffuse, supporting the idea that IFM exposed to low oxygen do not necessarily need to supply ATP for contraction. Furthermore, the fact that ETC complexes and ATP synthase are downregulated suggests a hypothetical scenario in which IFM are deactivated under low oxygen pressure but still functional and ready for an eventual surge upon renewed oxygen supply to avoid the risk of oxidative stress.

Our measurements of the matrix pH indicate that control IFM essentially lack a proton gradient. This is consistent with our findings of OXPHOS enzyme activities. The lower pH of the mitochondrial matrix suggests that the *pmf* has decreased but not necessarily been lost due to technical issues associated with the assay and measurement thresholds. Controlled decrease in transmembrane potential may serve as a mechanism to regulate the OXPHOS system, e.g. in parallel with uncoupling proteins. Indeed, cultured cells are not necessarily dependent on mitochondrial ATP production, as they can meet their energy through alternative metabolic pathways such as glycolysis. This is true even when they are transformed into Rho-zero cells, i.e. mtDNA-depleted cells and are therefore unable to form complexes I, III, IV, and V. Although mitochondria have been greatly remodeled not only functionally but also morphologically, Rho-zero mitochondria are still able to import proteins and they can generate sufficient transmembrane potential thanks to transporters located in the inner membrane (55). A lack of mtDNA quality, however, seems not to underlie the observed alteration in control IFM. Although we cannot rule out the possible presence of mtDNA breaks below the detection limit of Southern blot (Fig. 3B), the overall quality of mtDNA appeared to be comparable across all groups. A technical shortcoming in the implementation of the matrix pH measurement was that it was measured in frozen-thawed mitochondria. Although this is a known issue in coupled respiration, we believe our approach is supported by the emerging evidence that OXPHOS coupling is not significantly different in mitochondria from fresh and frozen-thawed samples (56, 57).

There are specific aspects related to ATP synthase in the heart. The enzyme can catalyze the synthesis and hydrolysis of ATP depending on the direction of the c-ring rotation. Regulation of such activities appears to be a protective mechanism and is critical during cardiac stress response. However, recent evidence suggests that inhibition of the hydrolysis activity by the mitochondrial ATP synthase inhibitor 1, IF1, (or pharmacologically) is also beneficial under certain conditions, e.g. when oxygen is scarce, as it maintains ATP levels. This again fits perfectly with our conclusion that low oxygen levels around IFM not only result in local OXPHOS shutdown but also in compensatory mechanisms of other upstream metabolic pathways. As mitochondria carry out and interconnect a number of pathways, these adaptions could reflect other layer of metabolic remodeling that IFM undergo from hypoxic states to normoxia. To clarify the exact reasons behind all these findings, however, more research is necessary.

Some of the results may appear contradictory, such as the seeming complete loss of ATP synthase activity (Fig. S3C) and its presence in the complexome dataset (Fig. 5A–D). Several aspects deserve consideration. A reasonable explanation for the observed discrepancy is the detergent used for solubilization, i.e. digitonin vs. DDM. While digitonin is a rather mild detergent that preserves high-molecular mass complexes such as respiratory SCs, DDM is a harsher detergent. Digitonin was specifically used for mitochondrial solubilization for complexome profiling analysis. Although mitochondrial ATP synthase is not part of a respiratory SCs, it forms dimers and oligomers, which were detected in the complexome dataset. These assemblies are not explicitly labeled in the BNE gels (Fig. 2A and B) because they overlap with other complexes and are present in much lower abundance, thus evading visualization and quantification in BNE gels. In addition, BNE and the complexome approaches have different detection thresholds, which may explain the seeming absence of ATP synthase in control IFM. Thus, MS-based complexome profiling offers much stronger support for our observations.

Naturally, it would be of great interest to test our concept in the hypertrophic heart. Chronic pressure overload, for example, leads to adaptive organ remodeling that begins with hypertrophy and gradually progresses to contractile failure (58). The mechanisms that determine the transition from a physiological to a pathophysiological response are still debated, but seminal work has shown that intracellular dedifferentiation and the initiation of autophagy may represent the tipping point toward contractile dysfunction (59). Mitochondria influence autophagic flux upon loss of mitochondrial ATP production, essentially linking OXPHOS and autophagy reciprocally (60–62). It is therefore reasonable to hypothesize that a lack of oxygen availability may be an important trigger for both OXPHOS shutdown and maladaptive remodeling. Also, the failing human heart shows substantial dedifferentiation particularly in the center of cardiomyocytes (63) possibly explaining how the terminally failing heart transforms into an “engine out of fuel” (64). In support of this notion, we previously rescued a mouse model of inflammatory cardiomyopathy associated with massive organ remodeling by hyperoxia treatment (65). To the best of our knowledge, however, the piglets included in this study were in good health and did not suffer from pressure overload and/or cardiac contractile dysfunction. Of note, decreased afterload also triggers a stress response that may be equally detrimental to cardiac function (66). We can only speculate, but the piglets kept under laboratory conditions may have simply led a sedentary lifestyle, which in turn would explain the decrease in IFM activity. Unwittingly, we may have studied young and healthy couch potatoes.

We questioned why the observed OXPHOS regulatory mechanism might have been overlooked in previous studies. Clearly, its unmasking required both a suitable animal model and a targeted experimental approach. In our case, we used piglets, which in physiology and anatomy are unlike more commonly used rodent models but very similar to humans (15), e.g. heart mass and stroke volume, but also heart rate of 60–70 beats per minute in pigs compared with 500–600 beats per minute in mice. The heart rate may have been of particular importance to unmask the mechanism because OXPHOS control mechanisms differ between fast and slow heart rate species (67–70). For example, F_1_F_O_-ATP synthase activity is downregulated specifically in slow heart rate hearts under certain conditions such as ischemia, presumably to prevent the ATP-consuming (reverse) mode of action (67–71). It is reasonable to assume that the downregulation of activity precedes the observed F_1_ subcomplex disintegration. Another advantage of the current study was the manipulation of tissue oxygenation by HBO ventilation. Furthermore, complexome profiling (32, 33) was used to determine the stoichiometry and composition of OXPHOS complexes at high spatial resolution, providing insights into the molecular mechanisms of OXPHOS control that may have previously escaped detection in whole tissue proteomic analyses. Nevertheless, caution is warranted when translating such findings from any animal model to clinical routine. For instance, yet undefined mechanisms might act differently in piglets and (diseased) humans and counteract the observed activation of OXPHOS. Also, the observed HBO-induced activation of OXPHOS may not be beneficial under all conditions, e.g. the assembly of all OXPHOS holoenzymes might not begin as stoichiometrically as it appeared to after 240 min of HBO ventilation. Instead, transient presence of a disproportionate ETC may produce excessive amounts of ROS, which could lead to damage and organ dysfunction. Equally, repeated (dis)assembly of OXPHOS enzymes without accompanying exercise could be harmful to patients and impede a healing process. Finally, HBO could induce toxic effects, which were not considered relevant and/or overlooked in the present study. Thus, the potential benefits and harms of HBO ventilation must be carefully weighed before use in patients.

Taken together, our results suggest that the catalytic subcomplex of the mitochondrial F_1_F_O_-ATP synthase, the F_1_ subcomplex, is of regulatory importance in the mammalian heart. Although mitochondrial biogenesis pathways also responded to improved tissue oxygenation, assembly from pre-existing proteins in the inner mitochondrial membrane allows for a rapid switch-off/switch-on mechanism essentially by recruiting mitochondria and their OXPHOS machinery rather than ramping up activities. This type of bioenergetic plasticity affects adaptation to increased workloads to the same extent as switching to resting state, when the amount of holo-assembled F_1_F_O_-ATP synthase is diminished by disintegration of the catalytic component, while a pool of F_O_/PS subunits in the inner mitochondrial membrane await rapid reassembly. The finding that this plasticity of OXPHOS complexes is oxygen-dependent could be of therapeutic importance in patients where ATP depletion otherwise may trigger a transition from cardiac hypertrophy to contractile failure. Our concept could be tested, for example, in clinics treating patients who have suffered diving accidents or acute acoustic trauma. If available, HBO treatment may also be used to support convalescence in patients recovering from long-term immobilization or ischemic insults.

## Materials and methods

### Study design

The aim of this study was to improve our understanding of how OXPHOS is controlled in distinct, presumably differently oxygenated, compartments of cardiomyocytes. We used anesthetized piglets ventilated under normobaric (control) and HBO conditions to manipulate tissue oxygenation. From these piglets, we isolated subpopulations of cardiac mitochondria from different cellular compartments. OXPHOS enzyme activities were measured, and comprehensive biochemical characterizations were performed. Experimental groups were randomly assigned, and sexes were mixed. The group sizes of at least three independent experiments and the statistical analyses are given in the respective figure legends.

### Porcine model of HBO ventilation

The study was approved by and performed according to the guidelines of the Committee of Animal Research at Martin-Luther-University Halle-Wittenberg and the Regierungspräsidium Halle, Germany. Briefly, a total of 32 piglets (age 3 months) were studied. The animals ranged from 29 to 35 kg in body mass. Prior to anesthesia piglets were sedated with azaperone (2 mg/kg i.m.). Total intravenous anesthesia was induced and maintained with 0.6–1.6 µg/kg/h sufentanil, 0.4 mg/kg/h midazolam, and 0.3 mg/kg/h pancuronium, via a peripheral intravenous line. Endotracheal intubation was done after sufficient depth of anesthesia was reached, and then piglets were ventilated in a pressure-controlled manner. The femoral artery was cannulated to allow intermittent blood sampling and continuous blood gas analyses, and to monitor arterial blood pressure, heart rate and temperature using the TrendCare sensor system (Philips Medizinsysteme, Böblingen, Germany). Intravenous lines for fluid management were established by insertion of a central venous catheter in a femoral vein. All parameters were recorded using the intensive care unit pilot software (CMA/Microdialysis, Solna, Sweden) and stored in a database for further analysis.

Study groups: (A) pressure-controlled ventilation under normobaric (control) conditions (Fig. S1); (B) pressure-controlled ventilation under HBO conditions (Fig. S2); 16 animals in each group. For HBO treatment, we used a hyperbaric chamber (Co. Sayers/Hebold, Cuxhaven, Germany) and the following standard treatment protocol: (1) Compression phase, the hyperbaric chamber was pressurized from ambient pressure (1 bar) to 2.4 bar within 10 min (Δp140 mbar/min), fraction of inspired oxygen concentration (FiO_2_) 0.21. (2) Isopression phase, lasted 240 min in total and the chamber pressure was constantly 2.4 bar, FiO_2_ = 1.0; two normoxic air-breaks 10 min each were carried out after 70 and 160 min during the isopression to minimize potential neurotoxic effects of HBO. (3) Decompression phase, the chamber was depressurized from chamber pressure (2.4 bar) to the ambient pressure within 5 min (−Δp280 mbar/min). Control piglets were continuously ventilated with a FiO_2_ of 0.3 under normobaric conditions. All animals were submitted to artificial hyperventilation, arterial hypertension, and hypotension after baseline period of 1 h. Between each interventional procedure was a stabilization period, in which all physiological parameters were allowed to normalize. The hyperventilation (60 min) was carried out by appropriate changes of the respirator settings and was controlled by the arterial partial carbon dioxide pressure (paCO_2_). The target paCO_2_ was 20 mmHg. Arterial hypertension was induced and maintained by norepinephrine infusion (5–10 µg/kg/min) for 30 min with a target mean arterial pressure (MAP) greater than 160 mmHg. Arterial hypotension was induced by continuous infusion of esmolol (1–1.5 mg/kg/min) and bolus injections of urapidil (5–25 mg) to achieve a MAP of 30 mmHg over a period of 60 min. Piglets were sacrificed by intravenous infusion of pentobarbital (10 mg/kg) followed by preparation of the heart to obtain the myocardial tissue and mitochondrial subpopulations.

### Isolation of left ventricular SSM and IFM

Two subpopulations of left ventricular cardiac mitochondria, i.e. subsarcolemmal mitochondria (SSM) and IFM, were isolated according to the protocol of Palmer et al. (14). In short, all steps were performed at 4°C. Immediately after excision, the heart was rinsed and transferred to ice-cold buffer A (220 mM mannitol, 70 mM sucrose, 5 mM MOPS, pH 7.4) and kept on ice until further processing. The left ventricle was isolated, cleaned, weighed, pieces snap-frozen, and stored in liquid nitrogen for further analyses (e.g. gene expression and proteome analysis), or minced using scissors in 10 mL/g of ice-cold buffer A+ (buffer A plus 2 mM EGTA and 0.2% BSA). The minced tissue was further homogenized using an ULTRA-TURRAX for 5 s, followed by homogenization using a Polytron tissue homogenizer (T-25, IKA-Werke, Staufen, Germany) for eight cycles at 500 rpm. The tissue homogenate was centrifuged at 500*×g* for 10 min at 4°C. The resulting supernatant (SN No. 1) essentially containing SSM was transferred to a new vial, and the resulting pellet (PT No. 1) was again homogenized and centrifuged as before. The resulting SN (No. 2) was combined with the SN (No. 1) from the first 500*×g* centrifugation step, and the resulting PT (No. 2) essentially containing IFM, nuclei and debris was kept on ice until further processing. The combined SN (No. 1 + 2) containing SSM was centrifuged at 3,000*×g* for 10 min at 4°C. The resulting SN (No. 3) was discarded and the resulting PT (No. 3) essentially containing SSM was washed with and resuspended in ice-cold mitochondrial storage buffer (100 mM KCl, 50 mM MOPS, 0.5 mM EGTA, pH 7.4) at a concentration of 25 mg mitochondrial protein/mL storage buffer and kept on ice. PT (No. 2) from the second 500*×g* centrifugation step essentially containing IFM, nuclei, and debris was treated with a broad-specificity protease from bacteria, Nagarse (5 mg per gram of tissue), in ice-cold buffer B (100 mM KCl, 50 mM MOPS, 2 mM EGTA, 0.2% essentially acid-free BSA, pH 7.4) and homogenized as before and then diluted with twice the volume of buffer B. The diluted homogenate was centrifuged at 500*×g* for 10 min at 4° C. The resulting SN (No. 4) essentially containing IFM was transferred to a new vial, and the resulting PT (No. 4) was again homogenized and centrifuged. The resulting SN (No. 5) was combined with the previous SN (No. 4) and the resulting PT (No. 5) was discarded. The combined SN (No. 4 + 5) was centrifuged at 3,000*×g* for 10 min at 4°C and the pellet essentially containing IFM was washed with and resuspended in mitochondrial storage buffer at a concentration of 25 mg/mL and kept on ice. Protein yield was quantified using conventional Bradford assay with BSA as external standard (Bio-Rad Laboratories, Munich, Germany).

### Measurement of mitochondrial enzyme activities

OXPHOS enzyme activities cI–cV and citrate synthase activity were quantified using freshly prepared SSM and IFM as described elsewhere (72). Enzyme activities cI + III and cII + III were assessed using freeze-thawed mitochondria. ACO activity was determined from freeze-thawed samples essentially as previously described (73). All assays were conducted at 30°C in an Ultrospec 3300 pro UV/Visible Spectrophotometer (Amersham Biosciences, Freiburg, Germany).

### Blue-native electrophoresis

BNE was adapted from previously published protocols (27, 28). Briefly, 400 µg of isolated mitochondria were resuspended in 40 µL solubilization buffer (50 mM imidazole, 1 M 6-aminohexanoic acid, pH 7.0) and solubilized with either 12 µL digitonin (20% w/v) or 10 µL DDM (10% w/v) and incubated for 1 h at 4°C under gentle rotation. Then, samples were centrifuged at 21,000*×g* for 40 min at 4°C. Following centrifugation, the protein yield of the SN was determined using the Pierce BCA protein assay (Perbio Science, Bonn, Germany). Three microliters of SERVA Blue (5%) (SERVA Electrophoresis GmbH, Heidelberg, Germany) dissolved in 6-aminohexanoic acid (500 mM) were added to 150 µg of solubilized mitochondrial protein. BNE was performed at 4°C and low voltage. Following BNE, gels were stained with Coomassie G-250 (0.25%) (Sigma-Aldrich, Taufkirchen, Germany), or individual bands were excised and separated in a second dimension using Tricine-SDS polyacrylamide gel electrophoresis and silver stained (30).

### Catalytic in-gel staining of OXPHOS enzymes

For catalytic in-gel staining, 30 µg of solubilized mitochondria from frozen-thawed samples were separated using BNE at 4°C and low voltage. Following the run, gels were washed twice with water before staining was performed according to a protocol described elsewhere (29). Briefly, for the determination of cI activity, the gel was incubated in a solution containing 0.1 mg/mL NADH, 2.5 mg/mL NBT (nitro blue tetrazolium chloride) and 2 mM Tris/HCl (pH 7.4) for 2–5 min with gentle agitation. The gels were fixed in 50% methanol, 10% acetic acid for 2 h, and immersed in distilled water. For the evaluation of complex V (cV) activity (ATP hydrolysis assay), the gel was placed in a solution containing 34 mM Tris, 270 mM glycine, 14 mM MgSO_4_, 8 mM ATP, and 0.2% Pb(NO_3_)_2_ (pH 7.8). Gels were incubated under gentle agitation for several hours. After staining was complete, the gel was washed multiple times in distilled water. The resulting stained gels were scanned and analyzed densitometrically using the AIDA Imaging software (Raytest, Berlin, Germany).

### Measurement of the mitochondrial matrix pH

Mitochondrial matrix pH was adapted from a protocol published by Kwast and Hand (26). Briefly, mitochondria from frozen-thawed samples (25 mg/mL) were incubated 20 min with 10 µM BCECF (2 h,7h-bis-(2-carboxyethyl)-5-carboxyfluorescein, Invitrogen, Karlsruhe, Germany) at 25°C. Samples were diluted 10-fold with ice-cold buffer (250 mM sucrose, 0.2 mM EDTA, 50 mM Tris/HCl, 0.2 mg BSA/ml, pH 7.8), centrifuged for 10 min at 9,000*×g* at 4°C and resuspended to obtain the stock suspension (25 mg/mL). 100 µL of the mitochondrial suspension were added to 3.9 mL reaction buffer (100 mM KCl, 80 mM sucrose, 20 mM MOPS, pH 7.0) and fluorescence was monitored using a Hitachi F2000 spectrofluorometer at 25°C. BCECF has an isosbestic point at 439 nm in the excitation spectra, so it can be used for ratiometric measurements. The pH-dependent ratio of the emission intensity was determined at 535 nm with excitation wavelengths of 500 and 450 nm. For calibration, 100 µL mitochondria were added to 3.9 mL lysis buffer (100 mM KCl, 20 mM MOPS, 0.07% Triton X-100) and fluorescence was monitored as described above. The pH value of the buffer varied by 0.2 pH units from pH6.0 to 8.5.

### Immunoblotting

Left ventricular heart tissue was snap-frozen in liquid nitrogen. The tissue was homogenized in extraction buffer (100 mM Tris/HCl pH8.0, 12.7 mM EDTA, 10% SDS) with 1 mL buffer per 100 mg tissue. Ten micrograms of protein or mitochondrial sample, as indicated in the text, were separated on 4–12% precast Bis–Tris NuPage gels (Invitrogen, Karlsruhe, Germany). Protein standards were used to calibrate migration. Proteins were transferred by semi-dry blotting to nitrocellulose membranes using the XCell II blot module. Blots were blocked with 5% (w/v) nonfat dry milk in 0.1% Tris-buffered saline/Tween20 (pH 7.5) and probed with antibodies. Anti-ATPase β subunit (MS503; 1/10,000), anticytochrome *c* (MSA06; 1/10,000) and anti-VDAC/Porin (MSA03; 1/10,000) were obtained from Mitosciences (Eugene, OR, USA); anti-ATPase α subunit (A-21350; 1/5,000) was obtained from Invitrogen (Karlsruhe, Germany).

### Quantification of transcript level

RNA was isolated from left ventricular heart tissue and mitochondrial subpopulations using TRIzol (Invitrogen, Karlsruhe, Germany). RNA was digested with RNase-free DNase and subsequently reverse transcribed to cDNA by standard protocol using the H-Minus First Strand cDNA Synthesis Kit (Fermentas, St. Leon-Roth, Germany). Expression levels were determined using a real-time PCR system (iQ5, Bio-Rad Laboratories GmbH, Munich, Germany) and Absolute SYBR Green Fluorescein premix (Thermo Scientific, Waltham, MA, USA). Gene-specific standards were used to calculate copy numbers. RNA from left ventricles was normalized to 18S rRNA and mitochondrial RNA was normalized to mg mitochondrial protein. The following primer pairs (5′-3′, forward/reverse) were used: *18S rRNA* agttggtggagcgatttgtc/ggcctcactaaaccatccaa (396 bp); *PPARGC1A* (*PGC1A*) taaagatgccgcctctgact/tgaccgaagtgcttgttcag (168 bp); *TFAM* ctgtggagggaacttcctga/gctgatcgaggtctttttgg (224 bp); *12S rRNA* ttaccaacccttgccaattc/acatgcttgaggagggtgac (283 bp); *ND1* catcctgacccctagccata/tgctcggattcataggaagg (296 bp); *COX1* agcgggtactggatgaactg/ttctgggtgtccgaaaaatc (364 bp); *ATP6* ttttattgcccccacgataa/attaatagggcgggtgttcc (390 bp); *ND6* aatccccaagcccattaaac/tggtggagttggttgtggta (266 bp); *CYTB* caacaacgcattcattgacc/aatatggatgctccgtttgc (228 bp).

### Southern blot

Southern blot was done to exclude mtDNA degradation and/or cleavage. Briefly, SSM and IFM were lysed in TENS buffer (50 mM Tris/HCl pH8.0, 40 mM EDTA pH8.0, 100 mM NaCl, 1% SDS) and incubated with RNase A (200 µg) at 37°C and Proteinase K (200 µg) at 56°C for 30 min each. DNA was extracted by phenol:chloroform:isoamyl alcohol (25:24:1) and subsequently precipitated by isopropanol. Pellets were dissolved in TE buffer (10 mM Tris pH8.0, 1 mM EDTA). mtDNA was linearized by *BsrG*I, separated using on a 0.8% agarose gel and blotted onto a Hybond XL nylon membrane (GE Healthcare Europe GmbH, Munich, Germany) essentially as described elsewhere (74). The membrane was probed against a 266 bp fragment corresponding to *ND6*. Probes were labeled by random priming with [α-32P] dCTP using the RediprimeTM II kit (GE Healthcare Europe GmbH, Munich, Germany). Sephadex G-50 Quick Spin columns (Roche Diagnostics GmbH, Mannheim, Germany) were used for radio-labeled DNA purification. Hybridized membranes were exposed to phosphor-imager screens and analyzed using the BAS 2500 Image Analysis System from FUJIFILM Life Science (Fuji film Europe GmbH, Düsseldorf, Germany).

### Complexome profiling and full proteome analysis

Detailed description of method and MS data has been deposited to the ProteomeXchange Consortium via the PRIDE partner repository (75–77). Dataset identifier for mitochondrial complexome analysis: PXD035554 (reviewer account details—username: reviewer_pxd035554@ebi.ac.uk, password: xihJzCiq). Dataset identifier for tissue proteome analysis: PXD047678 (reviewer account details—username: reviewer_pxd047678@ebi.ac.uk, password: kQXi2ALj).

### Statistical analyses

Data are the mean ± SEM with the repeats of each experiment stated in the figure legend. Statistical analyses were done using GraphPad Prism (v10) with *P* < 0.05 considered being statistically significant.

## Supplementary Material

pgae210_Supplementary_Data

## Data Availability

All study data are included in the article and/or supporting information. MS raw files and complete complexome and proteome data can be downloaded for extended analysis from the PRIDE database (75–77) (dataset identifier for the SSM and IFM complexomes: PXD035554; dataset identifier for the full proteomes: PXD047678).
