# Supplementary material for: Hyperbaric oxygen treatment reveals spatiotemporal OXPHOS plasticity in the porcine heart
Source: PNAS Nexus. 2024 May 30;3(6):pgae210. doi: 10.1093/pnasnexus/pgae210 (PMC11179111; doi:10.1093/pnasnexus/pgae210)
Supplement: pgae210_Supplementary_Data [file pgae210_supplementary_data.zip › PNASNEXUS-PNASNEXUS-2023-00907-TRR-s01.pdf]

Supporting Information for

Hyperbaric oxygen treatment reveals spatiotemporal OXPHOS plasticity in the porcine heart

Heidler, Cabrera-Orefice, Wittig et *al.*,

SI Appendix
Figures and Legends

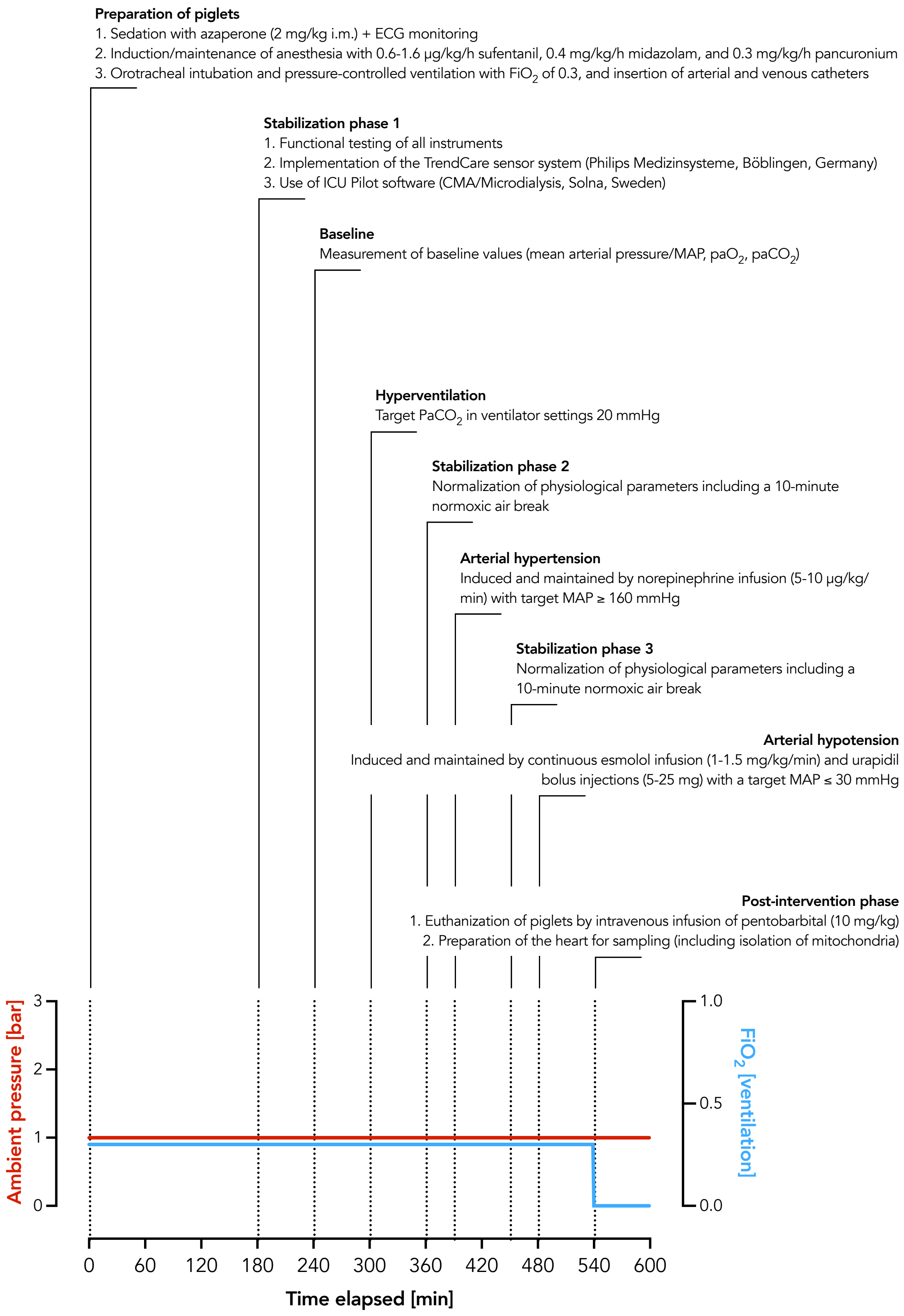

Preparation of piglets
1. Sedation with azaperone (2 mg/kg i.m.) + ECG monitoring
2. Induction/maintenance of anesthesia with 0.6-1.6 µg/kg/h sufentanil, 0.4 mg/kg/h midazolam, and 0.3 mg/kg/h pancuronium
3. Orotracheal intubation and pressure-controlled ventilation with FiO_2 of 0.3, and insertion of arterial and venous catheters
Stabilization phase 1
1. Functional testing of all instruments
2. Implementation of the TrendCare sensor system (Philips Medizinsysteme, Böblingen, Germany)
3. Use of ICU Pilot software (CMA/Microdialysis, Solna, Sweden)
Baseline
Measurement of baseline values (mean arterial pressure/MAP, paO_2, $paCO_2$)
Hyperventilation
Target $PaCO_2$ in ventilator settings 20 mmHg
Stabilization phase 2
Normalization of physiological parameters including a 10-minute normoxic air break
Arterial hypertension
Induced and maintained by norepinephrine infusion (5-10 µg/kg/min) with target MAP ≥ 160 mmHg
Stabilization phase 3
Normalization of physiological parameters including a 10-minute normoxic air break
Arterial hypotension
Induced and maintained by continuous esmolol infusion (1-1.5 mg/kg/min) and urapidil bolus injections (5-25 mg) with a target MAP ≤ 30 mmHg
Post-intervention phase
1. Euthanization of piglets by intravenous infusion of pentobarbital (10 mg/kg)
2. Preparation of the heart for sampling (including isolation of mitochondria)
Ambient pressure [bar]
3
2
1
0
FiO_2 [ventilation]
1.0
0.5
0.0
0
60
120
180
240
300
360
420
480
540
600
Time elapsed [min]

Supporting Figure 1. Experimental set-up of study group A, *i.e.*, control piglets ventilated at ambient conditions (relates to 'Porcine model of hyperbaric hyperoxic (HBO) ventilation' in Materials and Methods). Illustration of timeline and interventions as conducted.

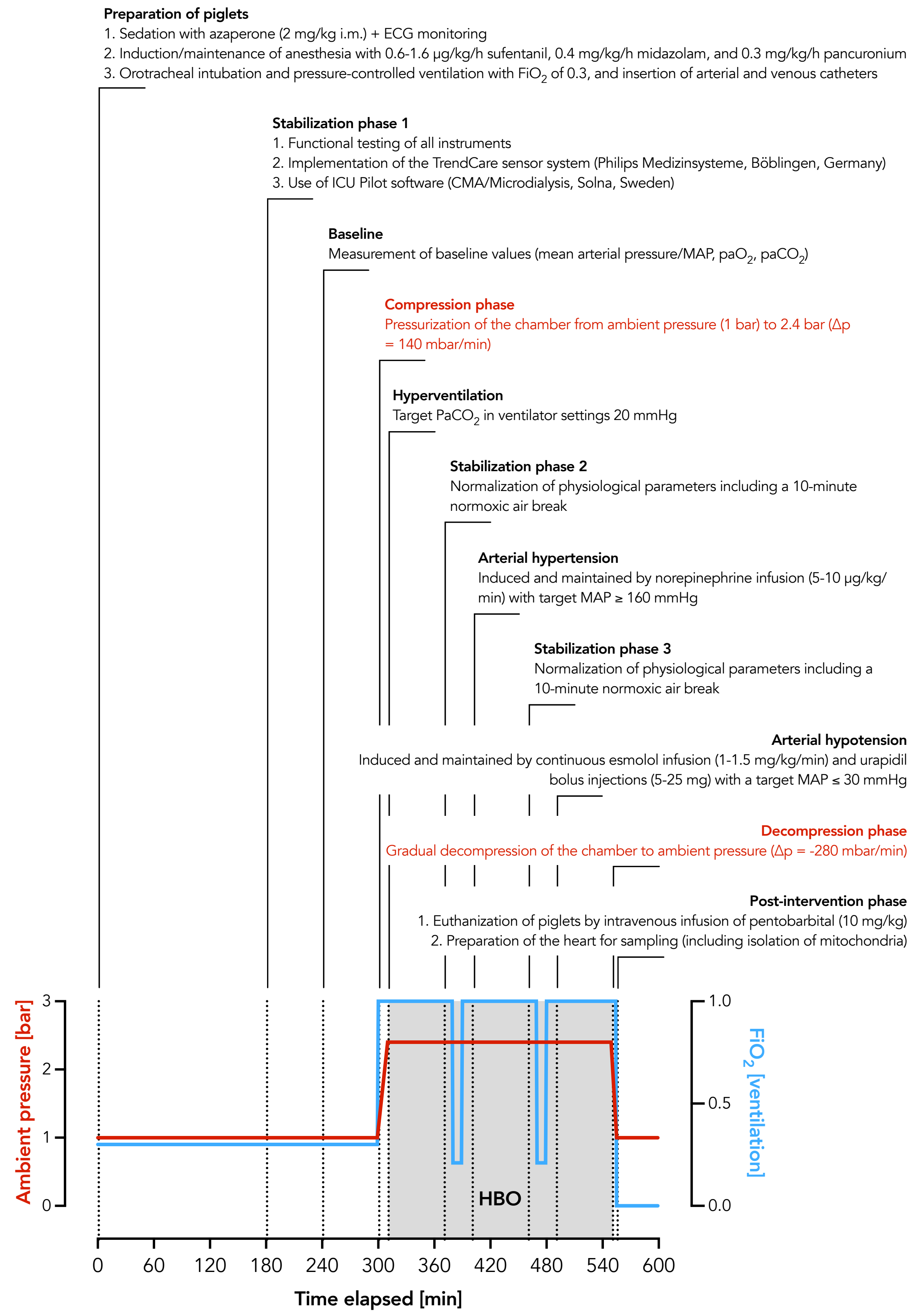
Preparation of piglets
1. Sedation with azaperone (2 mg/kg i.m.) + ECG monitoring
2. Induction/maintenance of anesthesia with 0.6-1.6 µg/kg/h sufentanil, 0.4 mg/kg/h midazolam, and 0.3 mg/kg/h pancuronium
3. Orotracheal intubation and pressure-controlled ventilation with FiO_2 of 0.3, and insertion of arterial and venous catheters
Stabilization phase 1
1. Functional testing of all instruments
2. Implementation of the TrendCare sensor system (Philips Medizinsysteme, Böblingen, Germany)
3. Use of ICU Pilot software (CMA/Microdialysis, Solna, Sweden)
Baseline
Measurement of baseline values (mean arterial pressure/MAP, paO_2, $paCO_2$)
Compression phase
Pressurization of the chamber from ambient pressure (1 bar) to 2.4 bar (Δp = 140 mbar/min)
Hyperventilation
Target $PaCO_2$ in ventilator settings 20 mmHg
Stabilization phase 2
Normalization of physiological parameters including a 10-minute normoxic air break
Arterial hypertension
Induced and maintained by norepinephrine infusion (5-10 µg/kg/min) with target MAP ≥ 160 mmHg
Stabilization phase 3
Normalization of physiological parameters including a 10-minute normoxic air break
Arterial hypotension
Induced and maintained by continuous esmolol infusion (1-1.5 mg/kg/min) and urapidil bolus injections (5-25 mg) with a target MAP ≤ 30 mmHg
Decompression phase
Gradual decompression of the chamber to ambient pressure (Δp = -280 mbar/min)
Post-intervention phase
1. Euthanization of piglets by intravenous infusion of pentobarbital (10 mg/kg)
2. Preparation of the heart for sampling (including isolation of mitochondria)
Ambient pressure [bar]
0
1
2
3
FiO_2 [ventilation]
0.0
0.5
1.0
HBO
0
60
120
180
240
300
360
420
480
540
600
Time elapsed [min]

Supporting Figure 2. Experimental set-up of study group B, *i.e.*, HBO-ventilated piglets (relates to 'Porcine model of hyperbaric hyperoxic (HBO) ventilation' in Materials and Methods). Illustration of timeline and interventions as conducted.

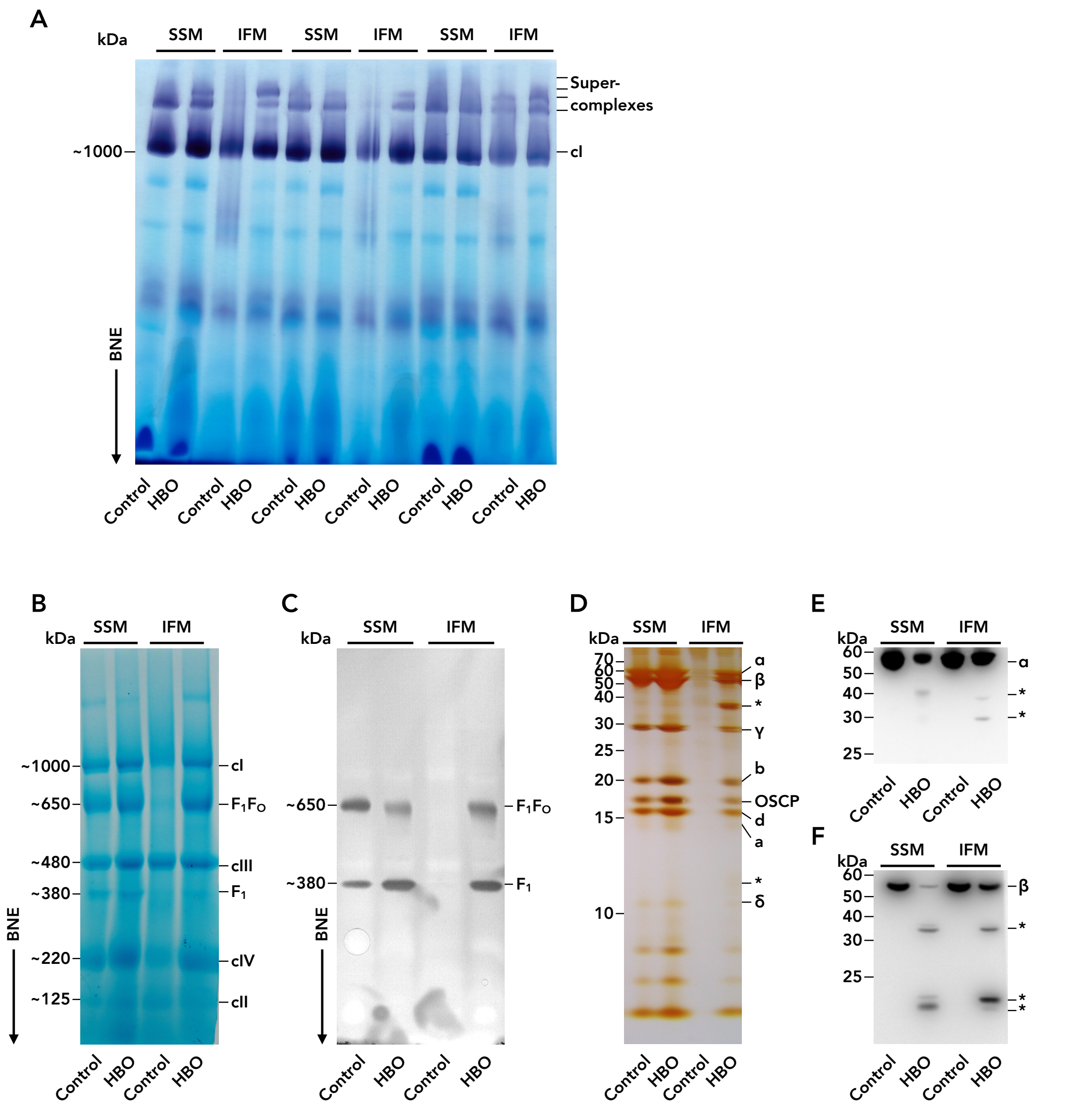

Supporting Figure 3. Expression pattern of OXPHOS complexes as well as activity and composition of F_1F_O-ATP synthase in DDM-solubilized SSM and IFM (relates to figures 1, 2, 4 and 5). (***A***) Gel showing catalytic in-gel staining of respiratory complex I (cI) from digitonin-solubilized and BNE-separated mitochondria. (***B***) First dimension of n-dodecyl-B-D-maltoside (DDM)-solubilized SSM and IFM from control and HBO-treated piglets as indicated using blue-native electrophoresis (BNE) followed by Coomassie staining. (***C***) First dimension BNE of samples as described for (***B***) followed by catalytic in-gel staining for ATPase activity. (***D***) Second dimension of excised F_1F_O-ATP synthase complexes from (***B***) using Tricine-SDS polyacrylamide gel electrophoresis for separation and silver stain for visualization individual subunits. (***E***) Western blot for subunit alpha of F_1F_O-ATP synthase (α). (***F***) Western blot for subunit beta of F_1F_O-ATP synthase (β). SSM, subsarcolemmal mitochondria; IFM, interfibrillar mitochondria; control, piglets ventilated at ambient conditions; HBO, piglets ventilated at hyperbaric hyperoxic conditions; cI-cIV, respiratory complexes I-IV; cV, mitochondrial F_1F_O-ATP synthase; *, unknown

proteins (potentially cleavage or degradation products) comigrating with F_1F_O-ATP synthase (***B***) and/or immunoreactive to antibodies directed against alpha or beta subunits of F_1F_O-ATP synthase (***E, F***).

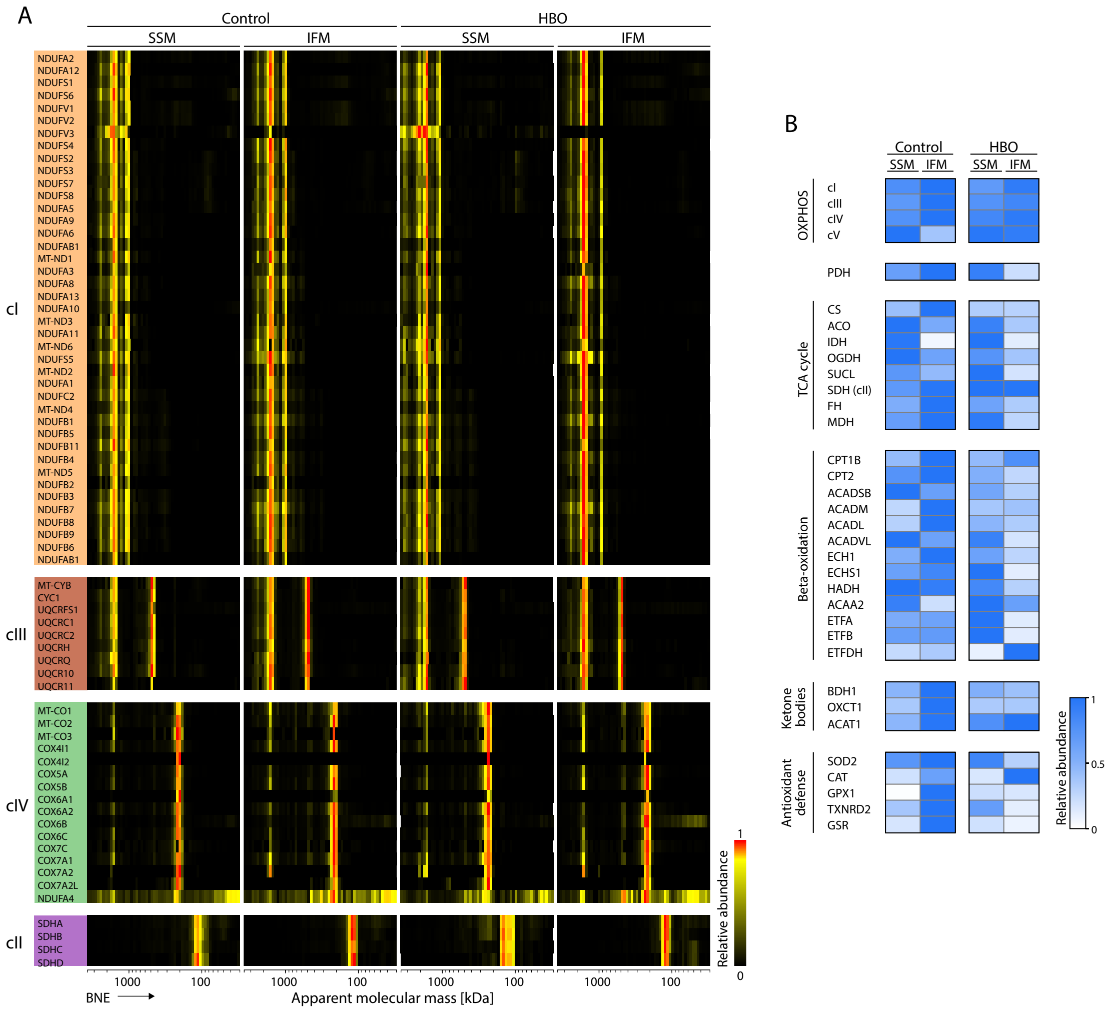

Supporting Figure 4. Extended complexome profiles of cardiac SSM and IFM (relates to figures 4 and 5). Mitochondrial subpopulations were isolated, and proteins were separated by blue native electrophoresis (BNE) followed by quantitative mass spectrometry analysis. The iBAQ values of each subunit were normalized using their maximal values across profiles. (***A***) Resultant relative abundance profiles are shown as heatmaps. Maximum appearance in red, up to 20% in yellow, black indicates that the proteins were not identified in the respective fractions. The list of all subunits identified for each respiratory complex are shown. (***B***) Mass spectrometry quantification of selected energy and ROS metabolism-related mitochondrial proteins of SSM and IFM from control and HBO-treated piglets. Protein abundance was calculated from complexome profiles as the area under the curve (AUC) as described in the main figure. Abundance values of individual components of multi-protein complexes were averaged. Data were normalized to the total protein abundance from each sample.
